# A Comprehensive Review of the Phytochemistry, Pharmacology and Other Applications of Euphorbiae Humifusae Herba

**DOI:** 10.3390/molecules30051094

**Published:** 2025-02-27

**Authors:** Jian Xiao, Hong Gu, Jiaqing Zhang, Yuqian Xue, Yunyi Chen, Weizhong Zhu, Hong Zhang, Boyi Fan, Wenli Wang

**Affiliations:** 1 School of Pharmacy, Nantong University, 9 Seyuan Road, Nantong 226019, China; 15902504768@163.com (J.X.); 15190810916@163.com (H.G.); 15380330612@163.com (J.Z.); 15651305816@163.com (Y.X.); 19502239732@163.com (Y.C.); zhuwz@ntu.edu.cn (W.Z.); 2 The Second Affiliated Hospital of Zhejiang Chinese Medical University, Hangzhou 310053, China; zhanghong_16@126.com

**Keywords:** Euphorbiae Humifusae Herba, pharmacological effects, chemical constituents, traditional use

## Abstract

Euphorbiae Humifusae Herba (EHH) is a globally distributed plant commonly utilized in traditional Chinese medicine (TCM) and health food within China. The dried aerial parts of EHH are well-recognized as health foods for the management of diarrhea and other intestinal diseases. Known for its therapeutic effects, such as heat-clearing, detoxification, blood cooling, hemostasis, dampness, elimination, and jaundice relief, EHH has yielded 197 bioactive compounds identified to date, including terpenoids, flavonoids, phenolic acids, tannins, alkaloids, sterols, lactones, coumarins, and other constituents, with flavonoids and terpenoids, highlighting its wide-ranging pharmacological properties and medicinal significance. Despite its popularity in research, limited systematic and comprehensive information has been provided on the EHH. Therefore, we provide an in-depth overview of EHH, covering its botanical characteristics, traditional uses, phytochemical composition, pharmacological properties, and additional applications. Furthermore, it addresses the current challenges and limitations in modern research on EHH, offering critical perspectives to guide future studies in this field.

## 1. Introduction

Euphorbiae Humifusae Herba (EHH) is the whole herb of *Euphorbia humifusa* Willd. or *Euphorbia maculata* L. in the Euphorbia family. Known by several names, including “Dijincao” (DJC), “Dijin”, and “Bandijin”, EHH is widely distributed across various provinces in China, with the exception of Hainan province. It holds significant importance in traditional Chinese medicine (TCM), where it has been used therapeutically for centuries [1,2].

Within TCM, EHH is traditionally valued for its effects in clearing heat and toxins, cooling blood, stopping bleeding, dispelling dampness, and reducing yellowing. Modern applications in Chinese medicine extend to its anti-inflammatory, hemostatic, liver-protective, and blood glucose-lowering properties [3]. Beyond its medicinal uses, EHH is consumed as a health food in China, and its extract is popular as a flavorful beverage in Korea [1,4,5]. These wide-ranging applications are attributed to its diverse chemical profile. To date, about 197 compounds have been identified in EHH, representing a broad spectrum of chemical groups, including terpenoids, flavonoids, phenolic acids and tannins, alkaloids, sterols, lactones, and coumarins, among others.

Currently, the Pharmacopeia of People’s Republic of China (2020) [2] lists four formulations containing EHH: Xiao’er Xieting Keli, Changyanning Pian, Changyanning Tangjiang, and Jidesheng Sheyao Pian. Notably, three of these formulations are primarily used to treat conditions such as diarrhea, dysentery, and both acute and chronic gastroenteritis, underscoring the significance of EHH as an essential component in TCM.

In recent years, extensive studies have examined the formulations, diverse compositions, pharmacological properties, and applications of EHH. Accordingly, this article aims to present a thorough and systematic review encompassing the botanical characteristics, ethnopharmacological uses, phytochemistry, pharmacological effects, and potential applications of EHH. This review is intended to serve as a valuable resource for advancing research and applications in the fields of medicine and chemistry.

## 2. Materials and Methods

The study design followed the guidelines of the PRISMA 2020 statement: an updated guideline for reporting systematic reviews [6].

### 2.1. Search Strategy

**Figure 1:**
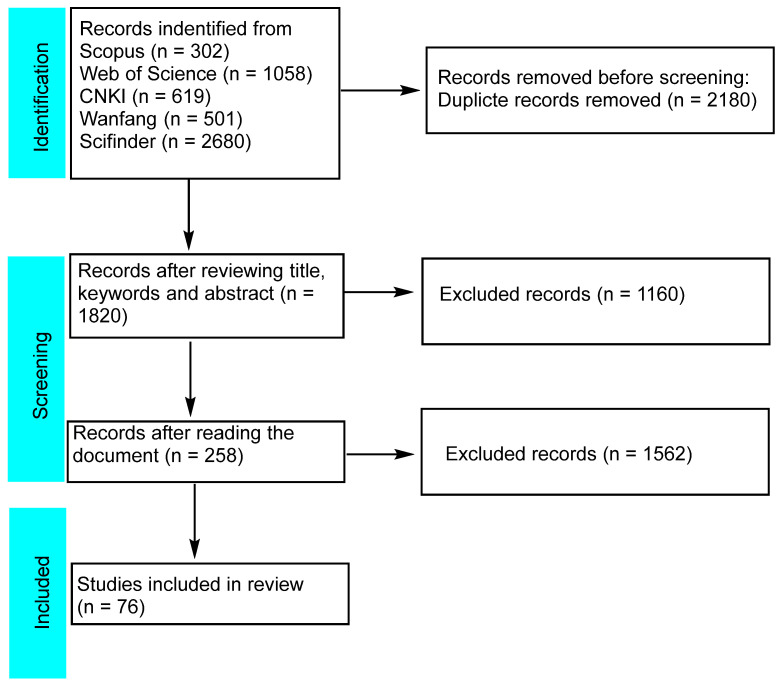
Search and selection of published articles.

Research data were sourced from reputable references, including the Pharmacopoeia of China and the Flora of China, as well as various online databases such as Scopus, CAS SciFinder, Web of Science, CNKI, and Wanfang Data. The databases were queried for articles published up to December 2024. The search terms utilized included “Euphorbiae Humifusae Herba,” “*Euphorbia humifusa* Willd”, “*Euphorbia humifusa*”, “*E.humifusa*”, “*Euphorbia maculata* L.”, “*Euphorbia maculata*”, “*E. maculata* L.”, “*Euphorbia maculata* ”, “Dijincao” (DJC), “Dijin”, and “Bandijin” (Figure 1).

### 2.2. Exclusion Criteria

The inclusion and exclusion criteria of evidence found in databases are listed below.

Inclusion criteria included the following:

1. Studies carried out in vitro, in animal clinical studies and used as food
2. Studies utilizing the fresh, dried or extracts of EHH
3. Phytochemical studies on EHH

Exclusion criteria included the following:

1. Duplication of data
2. The title or the abstract does not meet the inclusion criteria
3. The document does not meet the inclusion criteria

## 3. Botany

EHH is an annual herbaceous plant characterized by slender, unbranched roots measuring approximately 10–18 cm in length and 2–3 mm in diameter. The stem is prostrate, branching above the base, with tips that sometimes incline upward. The base is often tinged red or pinkish-red, reaching 20–30 cm in length and 1–3 mm in diameter, and bears soft or sparse hairs. The leaves are opposite, rectangular to elliptical, 5–10 mm in length and 3–6 mm in width, with a blunt, rounded tip and a slightly oblique, tapering base. They frequently display fine serrations on the upper edge, occasionally with a central oblong purple spot. The upper leaf surface is green, while the underside is light green, sometimes pinkish-red, with sparse fur on both sides. The petioles are very short, measuring 1–2 mm.

**Figure 2:**
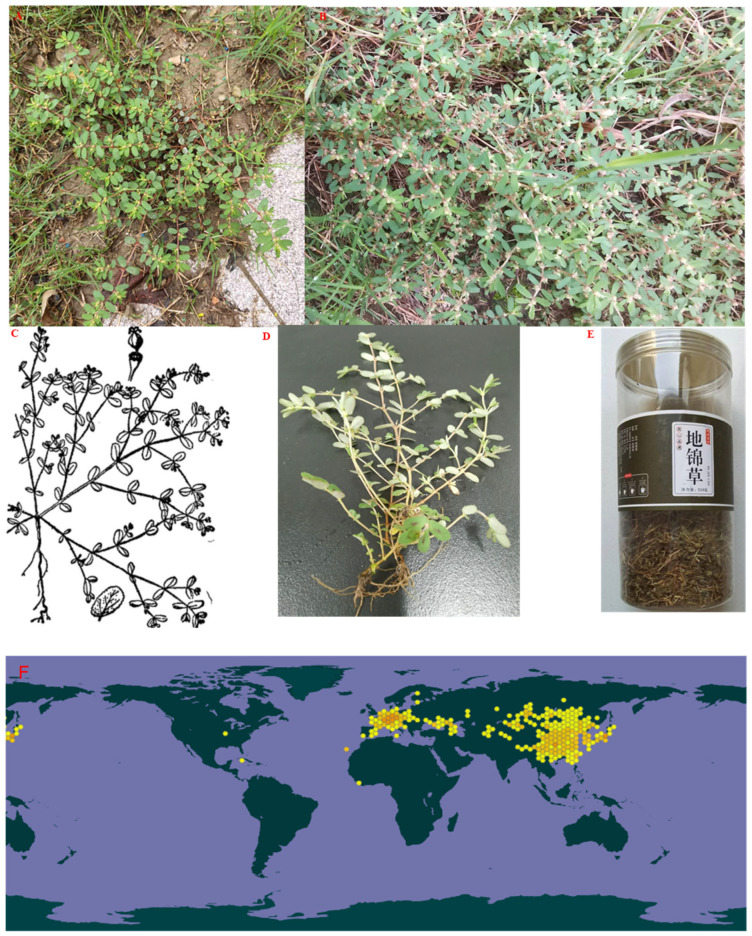

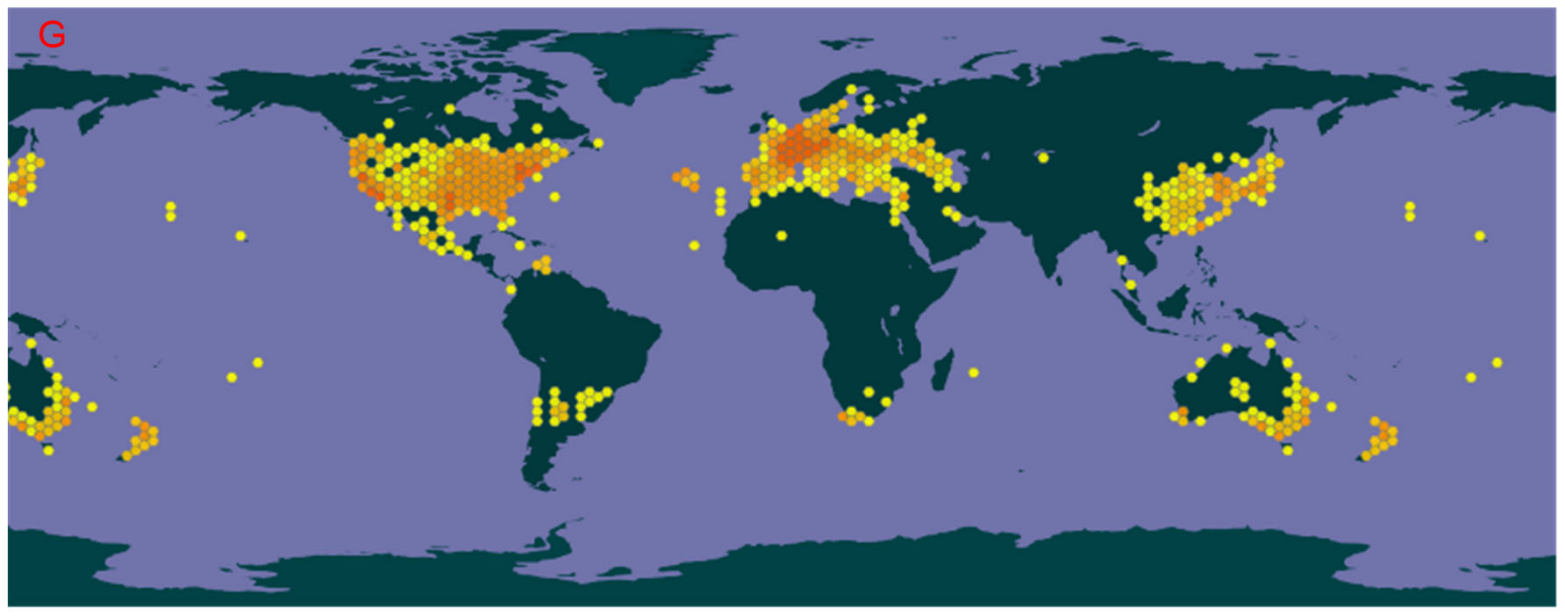
EHH plant photo. (**A**): *E. maculata* plant actual field photo; (**B**): *E. humifusa* Willd plant actual field photo; (**C**): The morphology of *E. humifusa* Willd (cited from http://www.iplant.cn (accessed on 28 December 2024)); (**D**): *E. maculata*; (**E**): The tea of EHH. (**F**): Geographical distribution of *E. humifusa* (Data from the Global Biodiversity Information Facility). (**G**): Geographical distribution of *E. maculata*.

Inflorescences appear solitary in the leaf axils, with a short stalk of 1–3 mm at the base. The gyro-shaped bracts are about 1 mm in both height and diameter, featuring four-lobed edges. The lobes are triangular, with four rectangular glands, each edged with white or pinkish-red appendages. Several male flowers extend outward, approximately matching the bract edge length, along with one outward-extending female flower with an ovary stalk reaching the bract edge. The capsule is triangular-ovate, about 2 mm long and 2.2 mm in diameter (Figure 2). Upon maturation, it divides into three fruit valves, leaving the style intact. Seeds are triangular-ovoid, approximately 1.3 mm long and 0.9 mm wide, gray, lacking transverse grooves or mounds on each edge. The flowering and fruiting period extends from May to October [7].

EHH is distributed throughout China, except in Hainan province, thriving in wild areas, roadsides, fields, sand dunes, beaches, and mountain slopes, especially in the regions north of the Yangtze River. It is also widely found in the temperate zone of Eurasia.

## 4. Traditional Uses

EHH has a rich history of use in TCM in China. Its medicinal properties were first documented in ancient texts such as “Jia You Ben Cao”, “Zheng Lei Ben Cao”, and the “Compendium of Materia Medica”, where the renowned physician. Li Shizhen described EHH as effective in treating abscesses, malignant sores, bleeding from cuts, and bloody dysentery, as well as for dispersing blood, stopping bleeding, and promoting urination. Other traditional texts, including “Jing Yan Fang”, “Qian Kun Sheng Yi”, and “Shi Yi De Xiao Fang”, also mention their therapeutic applications. Today, EHH remains commonly employed TCM. Classified in TCM as having a pungent and neutral taste, it primarily affects the liver and large intestine to promote heat-clearing, detoxification, blood cooling, hemostasis, dampness clearing, and yellowing reduction [2].

Beyond its role in TCM, EHH is used in Uyghur and Mongolian medicine, which it is applied to treat fungal infections like tinea (hand, body, foot, and versicolor) and psoriasis and is valued for cooling the blood and supporting liver health [8,9,10]. Additionally, EEH is also used in food preservation and other applications. Recent studies have shown that the ethanol extract can extend the freshness of strawberries, and its formulation, Dijincaokeli, is used in veterinary medicine to treat bacterial infections in chickens [11].

## 5. Phytochemistry

**Figure 3:**
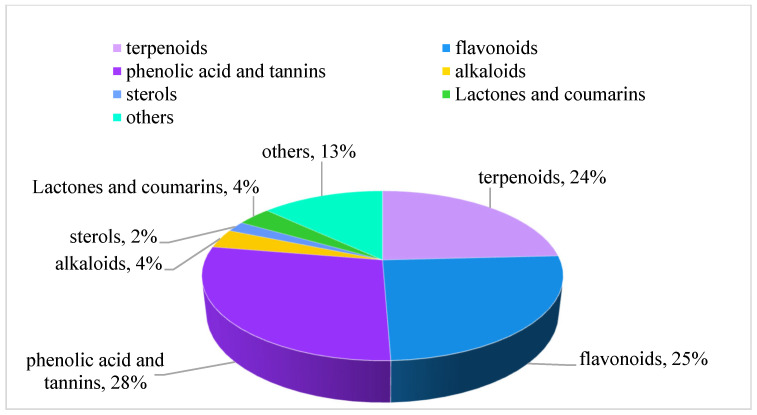
The proportion of chemical components in EHH.

To date, the literature reports the identification and isolation of 197 distinct compounds from EHH, representing a broad spectrum of chemical classes, including terpenoids, flavonoids, phenolic acids, tannins, alkaloids, sterols, lactones, coumarins, and other constituents. Among these, phenolic acids and tannins are the most prevalent, followed by flavonoids and terpenoids, while alkaloids, sterols, lactones, and coumarins are present in smaller quantities (Figure 3).

### 5.1. Triterpenoids

**Figure 4:**
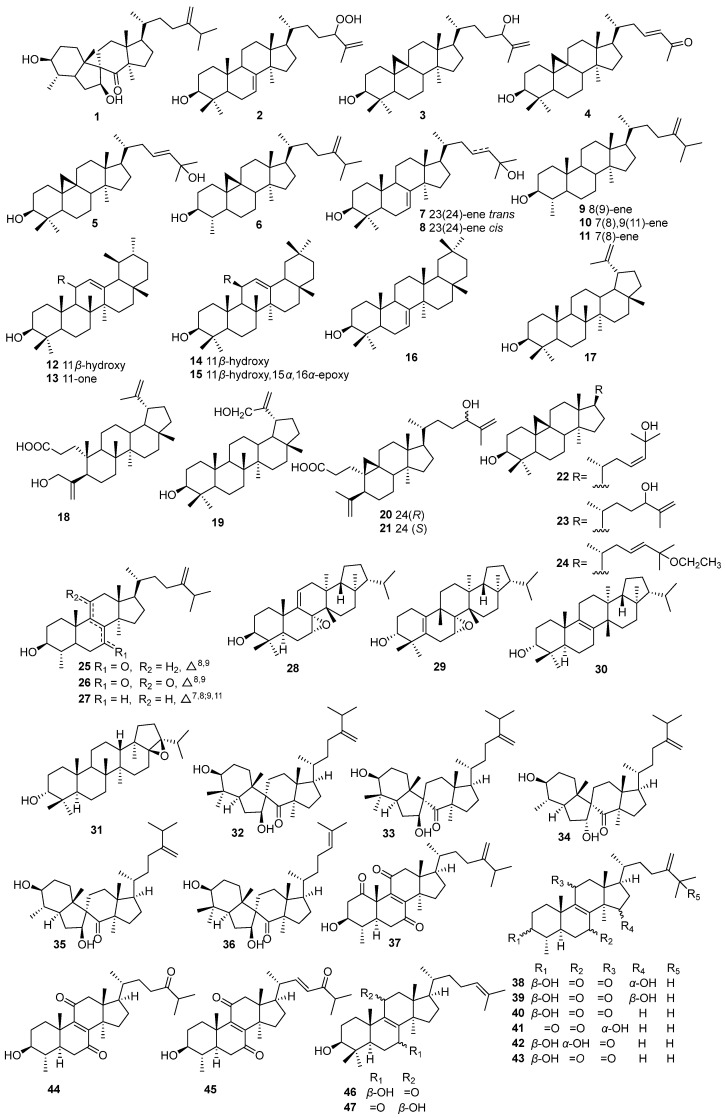
The structures of triterpenoids in EHH.

Triterpenes represent one of the primary active components in EHH and are categorized into four distinct types. Of these, triterpenoid compounds are more numerous, totaling 47 identified compounds (Figure 4, Table 1). Moreover, 17 triterpenes have been isolated from *E. maculata*, including such as: (3*S*,4*S*,7*S*,9*R*)-4-methyl-3,7-dihydroxy-7(8→9) *abeo*-lanost-24(28)-en-8-one (**1**) and 24-hydroperoxylanost-7,25-dien-3*β*-ol (**2**), 3-hydroxycycloart-25-ene-24-hydroperoxide (**3**), 3*β*-hydroxy-26-nor-9,19-cyclolanost-23-en-25-one (**4**), cycloart-23en-3*β*,25-diol (**5**), cycloeucalenol (**6**), (23*E*)-3*β*,25-dihydroxytirucalla-7,23-diene (**7**), (23*Z*)-3*β*, 25-dihydroxy-tirucalla-7,23-diene (**8**), obtusifoliol (**9**), 4*α*,l4*α*-dimethyl-5*α*-ergosta-7, 9(11), 24(28)-trien-3*β*-ol (**10**), gramisterol (**11**), urs-12-ene-3*β*, 11*α*-diol (**12**), neoilexonol (**13**), 12-oleanene-3*β*,11*β*-diol (**14**), (3*β*,15*α*,16*α*)-15,16-epoxy,olean-12-en-3-ol (**15**), multiflorenol (**16**), and lupeol (**17**) [12]. Additionally, 10 triterpenoids have been identified from *E. humifusa*, including 3,4-seco-lupa-4(23),20(29)-dien-24-hydroxy-3-oic acid (**18**), lup-20(29)-ene-3,30-diol (**19**), 24(*R*)-3,4-secocycloart-4(29),25-dien-24-hydroxy-3-oic acid (**20**), 24(*S*)-3,4-seco-cycloart-4(29),25-dien-24-hydroxy-3-oic acid (**21**), 23(*Z*)-cycloart-23-en-3,25-diol (**22**), 24(*S*)-cycloart-25-en-3,24-diol (**23**), 23(*E*)-cycloart-23-en-25-ethoxy-3-ol (**24**), 3-hydroxy-4,14-dimethyl-5-ergosta-8,24(28)-dien-7-one (**25**), 3-hydroxy-4,14-dimethyl-5-ergosta-8,24(28)-dien-7,11-dione (**26**), 3-hydroxy-4,14-dimethyl-5-ergosta-7,9(11),24(28)-trien (**27**) [13]. Further isolated from *E. humifusa* were (3*S*,5*R*,7*R*,8*S*,10*S*,13*S*,14*S*,17*R*,18*R*,21*R*)−7*α*,8*α*-epoxyfern-9(11)-en-3*β*-ol (**28**), (3*R*,7*R*,8*S*,9*S*,13*S*,14*S*,17*R*,18*R*,21*R*)−7*α*,8*α*-epoxyadian-5(10)-en-3*α*-ol (**29**), fern-8(9)-en-3*β*-ol (**30**), and 17*β*,21*β*-epoxyhopan-3*β*-ol (**31**) [14]. Additional compounds include spiromaculatols A–C, spiropedroxodiol, and spiroinonotsuoxodiol (**32**–**36**), euphomaculatoids A–E, 3*β*, 7*α*-dihydroxy-4*α*,14*α*-dimethyl-5α-ergosta-8,24 (28)-dien-11-one, and 3*β*-hydroxy-4*α*,14*α*-dimethyl-5*α*-ergosta-8,24 (28)-diene-7,11-dione (**37**–**43**), euphomaculatoids F-H (**44**–**46**), and 3*β*,11*β*-3,11-dihydroxylanosta-8,24-dien-7-one (**47**), derived from *E. maculate* [15].

### 5.2. Flavonoids

**Figure 5:**
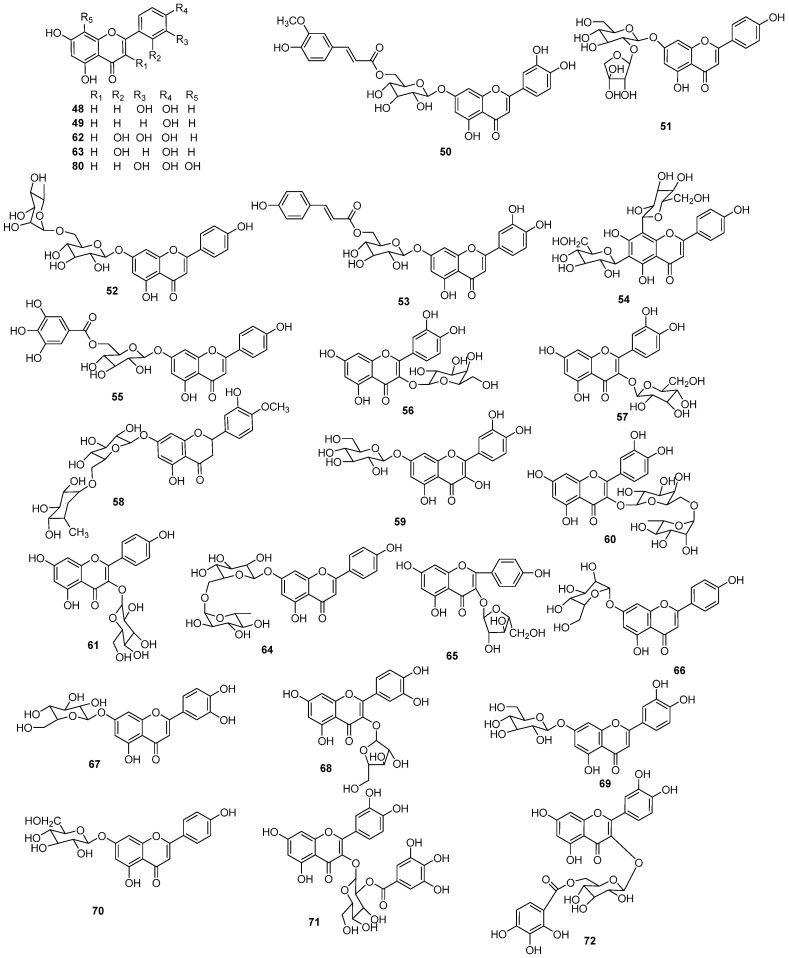

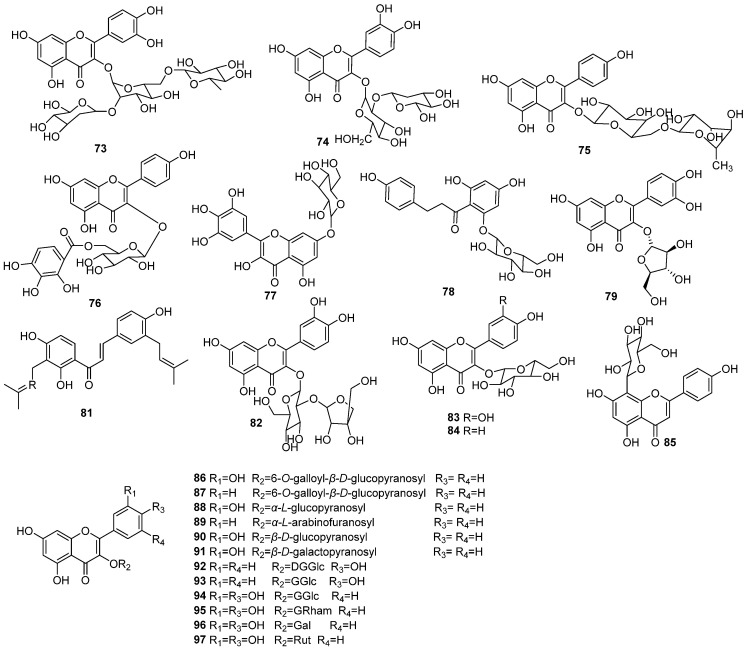
The structures of flavonoids in EHH (DGGlc: 2,3-digalloylglucosyl, GGlc: 2-galloylglucosyl, GRham: 2-galloylrhamosyl; Gal: galactosyl; Rut: rutinosyl).

The specific flavonoid composition in EHH includes various subclasses such as flavonoids, flavonols, flavanones, and dihydrochalcone, as well as their glycosides (Figure 5, Table 2) [3,4,16,17,18,19]. Ying Tian and colleagues have isolated 11 flavonoid compounds from *E. humifusa*, including luteolin (**48**), apigenin (**49**), luteolin-7-*O*-(6″-*O*-transferuloyl)-*β*-*D*-glucopyranoside (**50**), luteolin-7-*O*-(6″-*O*-coumaroyl)-*β*-*D*-glucopyranoside (**51**), apigenin-7-*O*-*β*-*D*-lutinoside (**52**), apigenin-7-*O*-*β*-*D*-apiofuranosyl(1→2)-*β*-*D*-glucopyranoside (**53**), 6,8-di-*C*-*β*-*D*-glucopyranosyl apigenin (**54**), apigenin-7-*O*-(6″-*O*-galloyl)-*β*-*D*-glucopyranoside (**55**), quercetin-3-*O*-*β*-*D*-galactoside (**56**), quercetin-3-*O*-*β*-*D*-glucopyranoside (**57**), and hesperidin (**58**) [15]. Xiaoying Wang has isolated quercetin-7-*O*-*β*-*D*-glucopyranoside (**59**), quercetin-3-*O*-*α*-*L*-rhamnosyl(1→6)-*β*-*D*-galactoside (**60**), and kaempferol-3-*O*-*β*-*D*-glucopyranoside (**61**) from *E. humifusa* [20]. Using polyamide chromatography, Rongzhi Li has identified quercetin (**62**) and kaempferol (**63**) from *E. humifusa* [21]. In 2008, Deng et al. found that isorhoifolin **(64)**, kaempferol-3-*O*-*α*-*L*-arabinoforanoside (**65**) [22]. Runhui Liu and colleagues have employed Sephadex LH-20 purification to isolate apigenin-7-*O*-glucoside (**66**), luteolin-7-*O*-glucoside (**67**), and quercetin-3-*O*-arabinoside (**68**) from *E. humifusa* [23]. Additional flavonoids identified in *E. humifusa* including luteolin-7-*O*-*β*-*D*-glucopyranoside (**69**), apigenin-7-*O*-*β*-*D*-glucopyranoside (**70**), quercetin-3-*O*-(2″,3″-di-*O*-galloyl)-*β*-*D*-glucopyranoside (**71**), quercetin-3-*O*-(6″-*O*-galloyl)-glucopyranoside (**72**), quercetin-3-*O*-{rhamnosyl-(1→6)-[xylosyl-(1→2)]-galactoside} (**73**), quercetin-3-*O*-*β*-*D*-xylosyl-(1→2)-*β*-*D*-glucopyranoside (**74**), nicotiflorin (**75**), kaempferol-3-*O*-(6″-*O*-galloyl)-*β*-*D*-galactoside (**76**), isomyricitrin (**77**) and phlorizin (**78**) [24]. Ahn et al. isolated quercetin-3-*O*-*α*-*L*-arabinofuranoside (**79**) from *E. humifusa* [3]. Four flavonoids, apigenin (**49**), quercetin (**62**), kaempferol (**63**), 8-hydroxyluteolin (**80**) and one prenylated chalcone paratocarpin E (**81**), were obtained from *E. humifusa* by Gao et al. [25]. Four flavonoid glycosides, quercetin-3-*O*-apiosyl (1–2) galactoside (**82**), quercetin-3-*O*-glucoside (**83**), astragalin (**84**), and vitexin (**85**), were isolated from *E. humifusa*. Isoquercitrin 6″-*O*-gallate (**86**), astragalin 6″-*O*-gallate (**87**), avicularin (**88**), juglanin (**89**), isoquercitrin (**90**), and hyperoside (**91**) were isolated from *E. maculata* by Nugroho [18]. Lastly, Luyen et al. have isolated 12 additional flavonoids from *E. humifusa*, including astragalin 2″,3″-*O*-digallate (**92**), astragalin 2″-*O*-gallate (**93**), isoquercitin 2″-*O*-gallate (**94**), quercitrin 2″-*O*-gallate (**95**), phlorizin (**78**), quercetin (**62**), hyperin (**96**), isoquercitrin (**90**), rutin (**97**), isomyricitrin (**77**), nicotiflorin (**75**), and astragalin (**84**) [4].

### 5.3. Phenolic Acids and Tannins

**Figure 6:**
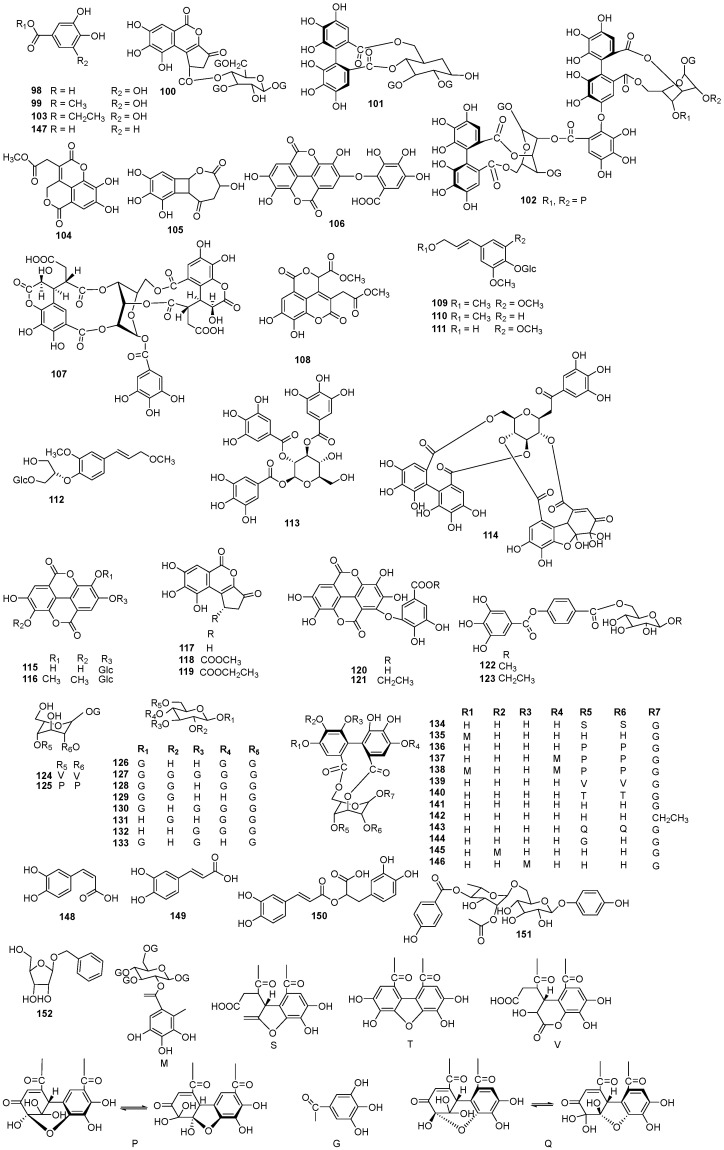
The structures of phenolic acids and tannins in EHH.

The 55 phenolic constituents of EHH include simple phenolic acids and ellagitannins (Figure 6, Table 3). Nugroho et al. have isolated gallic acid (**98**) and methyl gallate (**99**) from *E. humifusa* [18]. In 1994, Yoshida identified several tannins and phenolics, including 1,3,6-tri-*O*-galloyl-2-*O*-brevifolincarboxyl-*β*-*D*-glucose (**100**), ellimagrandin I (**101**), and excoecarianin (**102**) from a 70% acetone extract of *E. humifusa* using Ttoyptearl HW-40, MCI-gel, CHP-20P, among others [26]. Tian et al. have isolated ethyl gallate (**103**), phyllanthussin E methyl ester (**104**), humifusaone (**105**), and valoneaic aciddilactone (**106**) from *E. humifusa* in 2010 [16]. Yoshiaki and colleagues have isolated eumaculin E (**107**) from *E. maculata* [27]. In 2014, Luyen and colleagues fractionated several tannins and derivatives, including dehydropicrorhiza acid methyl diester (**108**), methylsyringin (**109**), methylconiferin (**110**), syringin (**111**), and sphaerophyside SC (**112**) from the methanolic extract of *E. humifusa* [4]. According to the literature, Isao isolated 1,2,3-tri-*O*-galloyl-*β*-*D*-glucose (**113**) from the aerial parts of *E. maculata* [28]. In 1997, Amakura et al. extracted granatin B (**114**) from an acetone extract of *E. maculata* [27]. Takashi et al. have isolated ellagic acid-4-*O*-*β*-*D*-glucopyranoside (**115**) from the above-ground parts of *E. humifusa* [26]. Deng et al. have obtained 3,3′-di-*O*-methyl ellagic acid-4-*O*-*β*-*D*-glucopyranoside (**116**) using silica gel column chromatography and Sephadex LH-20 from *E. humifusa* [22]. Luyen et al. have isolated brevifolin carboxylic acid (**117**) [4], and Tian and colleagues have obtained methyl brevifolin carboxylate (**118**), ethyl brevifolin carboxylate (**119**), sanguisorbicacid dilactone (**120**), 7″-ethyl-sanguisorbic acid dilactone (**121**), 1-*O*-methyl-6-*O*-p-digalloyl-*α*-*D*-glucopyranoside (**122**), 1-*O*-ethyl-6-*O*-p-digalloyl-*α*-*D*-glucopyranoside (**123**), chebulanin (**124**), and furosin (**125**) from *E. humifusa* [16]. Yoshida et al. have obtained 1,3,4,6-tetra-*O*-galloyl-*β*-*D*-glucose (**126**), euphormisin M3 (**127**), 1,2,4,6-tetra-*O*-galloyl-*β*-*D*-glucose (**128**), 1,2,6-tri-*O*-galloyl-*β*-*D*-glucose (**129**), 1,3,4,6-tetra-*O*-galloyl-*β*-*D*-glucose (**130**), 2,4,6-tri-*O*-galloyl-*D*-glucose (**131**), 3,4,6-tri-*O*-galloyl-*D*-glucose (**132**), and 1,3,6-tri-*O*-galloyl-*D*-glucose (**133**) were also extracted from 70% acetone extract of *E. humifusa* [26]. Using toyptearl HW-40 and Sephadex LH-20, Isao et al. have isolated euphormisin M2 (**134**), eumaculin A (**135**), geraniin (**136**), euphorbin B (**137**), euphorbin A (**138**), chebulagic acid (**139**), mallotusinin (**140**), corilagin (**141**), 1-*O*-ethyl-3,6-*O*-(*R*)-hexahydroxydiphenoyl-(1C4)-*β*-*D*-glucose (**142**), 1,3,4,6-tetra-*O*-galloyl-*β*-*D*-glucose (**143**), tercatain (**144**), eumaculin B **(145)**, and eumaculin D **(146)** from *E. maculata* [28]. Other compounds have been isolated, including protocatechuic acid (**147**), *cis*-caffeic acid (**148**), *trans*-caffeic acid (**149**) [29], rosmarinic (**150**) [30], euphorbinoside (**151**), and benzyl *β*-D-ribofuranoside (**152**) [4] were isolated from *E. humifusa*.

### 5.4. Alkaloids

**Figure 7:**
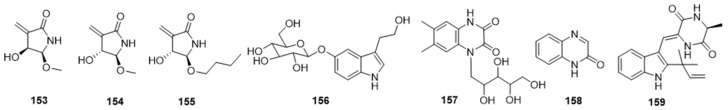
The structures of alkaloids in EHH.

Deng et al. have separated four alkaloids from *E. humifusa*: 5-*β*-methoxy-4*β*-hydroxy-3-methylene-*α*-pyrrolidinone (**153**), 5-*β*-methoxy-4*α*-hydroxy-3-methylene-*α*-pyrrolidinone (**154**), 5*β*-butoxy-4*α*-hydroxy-3-methylene-*α*-pyrrolidinone (**155**), and 3-(2-hydroxyethyl)-5-(1-*O*-glucopyranosyloxy)-indole (**156**) [22] (Figure 7, Table 4). Tian et al. have separated 1-(2′,3′,4′,5′-tetrahydroxypentyl)-6,7-dimethyl-guinoxaline-2,3-(1H,4H)-dione (**157**), from *E. humifusa* [16]. Two other alkaloids have also been separated from *E. humifusa* called uinoxadione (**158**) [26] and (−)-neoechinulin A (**159**) [29].

### 5.5. Sterols

**Figure 8:**
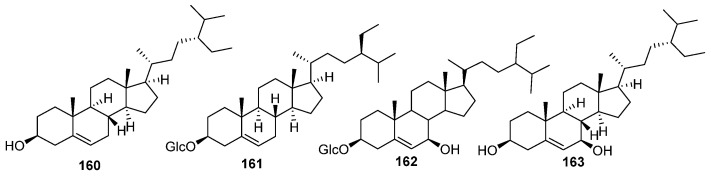
The structures of sterols in EHH.

The sterols identified from *E. humifusa,* including *β*-sitosterol (**160**), *β*-daucosterol (**161**), stigmaster-5-ene-3-*O*-(6-linoyl-114yl)-*β*-*D*-glucopyranoside (**162**) and 7*β*-hydroxy-sitosterol (**163**) [24] (Figure 8, Table 5).

### 5.6. Lactones and Coumarins

**Figure 9:**
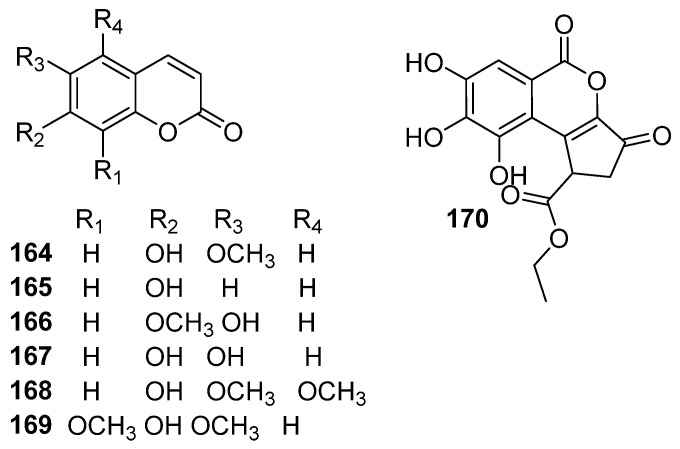
The structures of lactones and coumarins in EHH.

Several lactones and coumarins have been isolated from *E. humifusa*, including scopoletin (**164**) [24], umbelliferone (**165**) [23], 7-methoxy-6-hydroxyl-coumarin (**166**) [13], esculetin (**167**), 5-methoxyscopoletin (**168**), isofraxidin (**169**) [29], and ethyl brevifolincarboxylate (**170**) [13] (Figure 9, Table 6).

### 5.7. Other Compounds

**Figure 10:**
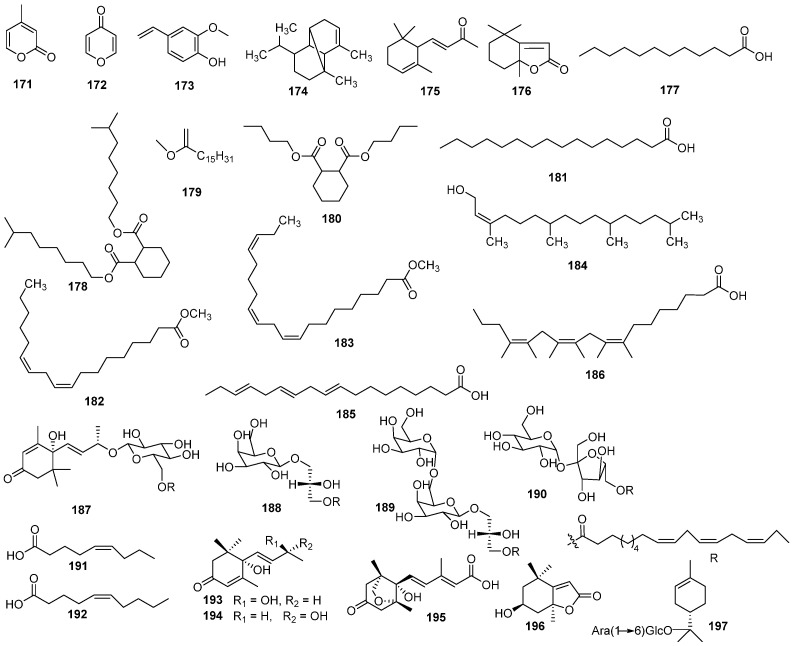
The structure of other compounds in EHH.

Previous analyzing the volatile components of EHH have identified ketones (32.33%), acids (25.32%), and esters (14.55%) as their primary constituents [31] (Figure 10, Table 7). Additional compounds identified in this plant include *α*-pyrone (**171**), *γ*-pyrone (**172**), 2-methoxy-4-vinylphenol (**173**), desogestrel (**174**), *α*-ionone (**175**), dihydroactinidiolide (**176**), lauric acid (**177**), isononyl phthalate (**178**), methyl hexadecanoate (**179**), dibutyl phthalate (**180**), palmitic acid (**181**), methyl linoleate (**182**), 9,12,15-octadecatrienoic acid, methyl ester (**183**), phytol (**184**), linoleic acid (**185**), and *α*-linolenic acid (**186**). From the whole plant of *E. humifusa*, four fatty glycosides have been isolated: humionoactosides A (**187**), (2*S*)-3-*O*-octadeca-9*Z*,12*Z*,15*Z*-trienoylglyceryl-*O*-*β*-D-galactopyranoside (**188**), ingerglycolipid A (**189**), and 6′-*O*-linolenoylsucrose (**190**) [32]. Other compounds obtained (5*Z*)-Nonenoic acid (**191**), (5*Z*)-undecenoic acid (**192**), corchoionol C (**193**), vomifoliol (**194**), (−)-phaseic acid (**195**), and isololiiolide (**196**) were also isolated from *E. humifusa* [29]. (4*S*)-*α*-terpineol 8-*O*-[*α*-L-arabinopyranosyl-(1→6)-*β*-D-glucopyranoside] (**197**) was isolated from *E. humifusa* [4]. *E. humifusa* also contains inorganic trace elements such as K, Mg, Ca, Na, Fe, Mn, Zn, and Cu [33].

## 6. Pharmacological Effects

**Figure 11:**
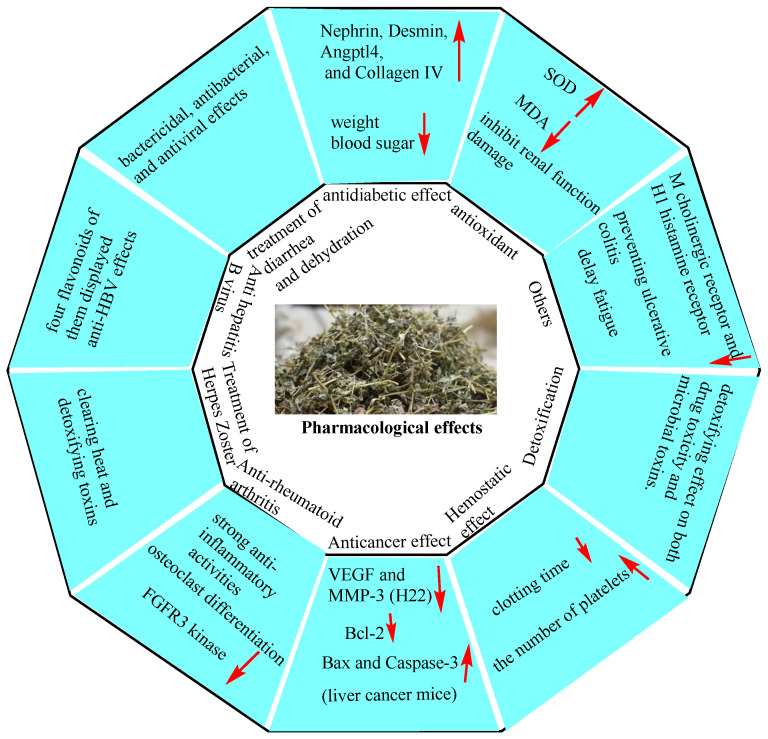
EHH has a wide range of pharmacological activities.

EHH has a sweet taste and is considered relatively mild in its medicinal action. Traditionally, EHH is used to clear heat and detoxify, cool the blood, stop bleeding, alleviate dampness and jaundice, and treat diarrhea. Recent research has revealed that EHH possesses multiple pharmacological activities, including hypoglycemic, antioxidant, anti-diarrhea, antibacterial, antiviral, anti-allergic (for skin conditions), anti-rheumatoid arthritis, antiemetic, detoxifying, anticancer, and chronic urticaria treatments. These pharmacological effects align with its traditional therapeutic applications (Figure 11).

### 6.1. Hypoglycemic Effect (Diabetes and Diabetic Nephropathy)

Diabetic kidney disease (DKD), a prevalent microvascular complication of diabetes, poses a serious health threat and is the primary cause of end-stage renal disease. In a 2023 study, Li et al. investigated the effects of *E. humifusa* on DKD by dividing specific pathogen-free (SPF) rats into three groups: a normal group, a DKD group, and a DKD group treated with *E. humifusa*. Findings have revealed that the expression levels of Nephrin, Desmin, Angptl4, and Collagen IV in podocytes are significantly higher in the *E. humifusa*-treated DKD group than in the untreated DKD group. This increase inhibits podocyte transdifferentiation, resulting in reduced proteinuria and improved renal function in DKD rats, indicating that EHH holds therapeutic potential for diabetic nephropathy [34].

Diabetes mellitus (DM) arises from a combination of genetic and environmental factors, leading to insufficient insulin secretion, insulin resistance, and consequential metabolic disturbances in glucose, protein, fat, water, and electrolyte balance. Type II diabetes constitutes the majority of cases [35]. In a study using KK-Ay mice on a high-fat diet, the mice are separated into several groups: a metformin group, a model group, low-dose and high-dose *E. humifusa* groups, and a control group regular diet. A few weeks later, enzyme-linked immunosorbent assay (ELISA) is conducted to measure serum insulin, TNF-*α*, IL-6, adiponectin, and leptin levels across the groups. The results show that *E. humifusa* effectively reduces body weight and fasting blood glucose in mice, alleviating pancreatic islets stress, thereby demonstrating a notable hypoglycemic effect [36,37]. Previous studies further support that polysaccharides, saponins, flavonoids, alkaloids, and phenols in EHH contribute to its blood glucose-lowering effects [38]. Collectively, these findings underscore *E. humifusa* as a promising agent for blood sugar regulation.

### 6.2. Antioxidant Properties

The total flavonoids from EHH have shown potential as feed additives in broiler chicken diets, enhancing immune function, antioxidant capacity, and intestinal microecological balance, thereby improving overall production performance [39]. Additionally, *E. humifusa* has been found to increase superoxide dismutase (SOD) activity in mice, a critical enzyme in oxidative and antioxidative processes [40]. It also inhibits malondialdehyde (MDA) production, thus protecting cell membranes from oxidative damage.

In studies involving a rat model of renal ischemia-reperfusion injury, treatment with *E. humifusa* significantly decreases MDA levels and increases SOD activity compared to untreated groups, indicating its ability to mitigate renal function damage caused by ischemia-reperfusion group through antioxidative free radical effects [41]. Further research has demonstrated a marked reduction in plasma lipid peroxides following treatment with *E. humifusa* water extract, indicating its potential for reducing oxidative stress [42]. In aging mice, the total flavonoids of *E. humifusa* increase telomerase content and SOD activity in testicular and brain tissues while reducing MDA levels, contributing to its antioxidant properties [43]. In summary, these studies affirm the potent antioxidant effects of EHH.

### 6.3. Treatment of Diarrhea and Dehydration

Fungal, viral, and bacterial infections are among the most common causes of diarrhea, and *E. humifusa* contains various active compounds with bactericidal, antibacterial, and antiviral properties. Domestic research has shown that [15] flavonoids, tannins, and phenolic acids in *E. humifusa* are the primary active ingredients for treating diarrhea. Studies indicate that *E. humifusa* is effective in treating recurrent ulcerative colitis [44] and has an inhibitory effect on small intestine peristalsis, making it highly effective in managing diarrhea and dehydration [45].

The extract of *E. humifusa* has demonstrated strong antimicrobial activity in vitro. For instance, it showed a significant inhibitory effect against 20 common pathogenic bacteria, while concentrations of 0.005–1.25 mg/mL exhibit bactericidal effects. Notably, compounds such as gallic acid and quercetin isolated from *E. humifusa* have also shown potent antibacterial activity [46]. The ethanol extract of *E. humifusa* has proven effective against dysentery bacteria, typhoid and paratyphoid bacteria, *Proteus* species, pathogenic *Escherichia coli*, and others [47].

In TCM, EHH is traditionally used for heat-clearing and detoxification, exhibiting detoxifying effects against various microbial toxins. Its antiviral effect may stem from its ability to alter the ultrastructure of endotoxins, rendering them non-toxic [48].

### 6.4. Anti-Hepatitis B Virus (HBV) Effects

HBV has been a major global health threat since the 1960s, with significant implications for human life and health. According to the World Health Organization (WHO), about 2 million people die from liver disease every year worldwide, including about 1 million deaths from complications of liver cirrhosis. China accounts for about 11 percent of the total deaths from live cirrhosis globally. According to data from 2015, China has the largest number of patients with chronic hepatitis B (CHB), about 74 million. In the development of CHB, compensated cirrhosis, about 4–12% of patients develop decompensated cirrhosis every year [49]. However, it is necessary to find the active constituent from a natural source.

In research conducted by Tian, 38 compounds have been isolated from *E. humifusa*, with four flavonoids demonstrating anti-HBV effects in vitro. This marks the first discovery of natural products with significant anti-HBV activity in vitro, presenting promising candidates for anti-HBV drug development [16].

### 6.5. Treatment of Herpes Zoster (HZ)

HZ, an acute skin disease caused by the varicella-zoster virus (VZV), arises when it invades the body [50]. In TCM, HZ is thought to result from damp-heat and fire-toxin imbalance. EHH is traditionally used to dispel external toxins through clear-heat and detoxifying actions, as well as to alleviate damp-heat.

The mechanism of HZ is linked to damp-heat accumulation and stagnation of heat in the qi and blood, causing congestion in blood vessels; *E. humifusa* is believed to regulate the qi clear heat and promote diuresis, helping to relieve these symptoms. According to Ben Cao Hui Yan, EHH is used to cool the blood and disperse blood, detoxify, and stop dysentery. It is particularly effective in clearing bleeding veins and detoxifying sores.

Modern pharmacological studies support these traditional uses, showing that some compounds isolated from *E. humifusa* exhibit anti-inflammatory properties, potentially contributing to its heat-clearing and detoxifying effects [4]. These findings suggest that EHH may offer therapeutic benefits in managing HZ.

### 6.6. Anti-Rheumatoid Arthritis (RA)

RA is a prevalent chronic autoimmune disease that affects multiple systems and is associated with prolonged disability. While RA can manifest at any age, it is most commonly diagnosed in young adults, with a prevalence rate reaching between 0.2% and 0.4%, significantly diminishing patients’ quality of life. Extensive studies have investigated the chemical constituents of *E. humifusa*, with particular emphasis on quercetin and kaempferol for their therapeutic effects on RA [51]. Quercetin, a natural flavonoid isolated from EHH, primarily alleviates RA symptoms by inhibiting processes like angiogenesis, synovial hyperplasia, inflammatory factor infiltration, and neutrophil extracellular trap formation. The study analyzed the therapeutic role of quercetin in collagen-induced arthritis in C57BL/6 mice. The animals were allocated into five groups that were subjected to the following treatments: negative (untreated) control, positive control (arthritis-induced), arthritis + methotrexate, arthritis + quercetin, and arthritis + methotrexate + quercetin [52]. Additionally, it modulates the balance between ruptured/osteoblasts and influences Th17/regulatory T cell dynamics, involving mediators such as TNF-*α*, MCP-1, IL-6, IL-1*β*, IL-17, IL-10, NF-*κ*B, CXCL1, CXCL5, LTB4, TGF-*β*, as well as various immune cells like neutrophils and macrophages [52,53,54,55,56,57,58]. Kaempferol, another flavonoid isolated from EHH, on the other hand, exerts anti-RA activity mainly through inhibition of FGFR3 kinase activity. Firstly, The MTT assay to investigate the cytotoxicity of kaempferol resulted in no cytotoxicity in murine CD^4+^ T-cells up to 25 μM of kaempferol. Next, the research investigated whether kaempferol suppressed inflammation and joint destruction in an experimental RA murine model. One group of mice was intraperitoneally injected with 2 mg/kg of kaempferol three times a week after type II collagen-boosting immunization, and the other group was only injected with the vehicle [59]. The results showed that it could reduce osteoclast differentiation both in vivo and ex vivo. and downregulate osteoclast markers such as tartrate-resistant acid phosphatase, integrin *β*3, and MMP9 [59]. Furthermore, kaempferol demonstrates potent anti-inflammatory effects by modulating pathways including MAPK, PKC, and PI3K, as well as inflammatory mediators IL-1, IL-8, IP-10, PGE2, IL-2, TNF-*α*, NF-*κ*B, and AP-1 [60,61], which are critical in RA pathogenesis. These findings suggest that *E. humifusa* may possess potential anti-RA properties.

### 6.7. Anticancer Effects

EHH has demonstrated inhibitory effects on liver cancer cell growth, potentially due to decreased expression of VEGF and MMP-3 in H22 tumor-bearing mice. In research conducted by Zou, mice are divided into five groups: model control, positive control, and three treatment groups receiving (high 264 mg/kg·d), medium (132 mg/kg·d), and low (66 mg/kg·d) doses of EHH. The treatment is administered over 14 days, after which tumor weight and size are measured, and histopathological examination of tumor tissues is conducted under an optical microscope. Immunohistochemistry reveals significantly reduced VEGF protein expression in the high-dose group (0.160 ± 0.004) compared to the model control group (0.228 ± 0.020), *t* = 5.011, *p* < 0.001. Similarly, MMP-3 expression is markedly lower across all EHH dose groups (0.316 ± 0.062, 0.303 ± 0.057, and 0.302 ± 0.058) than in the control group (*t* = 6.322, 6.845, and 6.534, *p* < 0.001) [62,63].

Further research suggested that EHH inhibits liver cancer cell growth by enhancing antioxidative capacity, reducing Bcl-2 expression, and upregulating Bax and Caspase-3 expression in transplanted liver tumors [64]. Another proposed mechanism involves the suppression of tumor angiogenesis, potentially through activation of the NF-*κ*B/VEGF signaling pathway [65]. In a separate study by Geng, administration of a high dose of DJC (water extract of DJC) significantly increased p19ARF in tumor tissues [66]. Xie’s study has reported a total effectiveness rate of 91% in treating 290 lung cancer patients with *E. humifusa* [49], while Jiang’s research has indicated that *E. humifusa* can inhibit Hela cell proliferation, potentially by inducing apoptosis [67]. Collectively, these findings underscore the substantial anticancer potential of *E. humifusa*, suggesting it may play a valuable role in cancer therapy.

### 6.8. Hemostatic Effects

Research on the hemostatic properties of *E. maculate* has demonstrated a marked ability to significantly reduce clotting time, thereby showing a strong hemostatic effect [68]. Similarly, studies on *E. humifusa* have revealed that it can swiftly elevate platelet counts, contributing to effective hemostasis [69]. Given these properties, *E. humifusa* shows potential as a treatment for various bleeding disorders, dental bleeding, acute hemorrhagic necrotizing enteritis, and other acute bleeding conditions.

### 6.9. Detoxification

*E. humifusa* has been shown to mitigate the severe organ damage induced by the chemical hexachlorocyclohexane, effectively protecting the heart, liver, spleen, kidneys, and other organs in animals, demonstrating a greater protective effect than vitamin C [70]. Additionally, microbial toxins, which lead to various symptoms and organ damage during infections, are notably countered by DJC tinctures at 100%, 50%, and 25% concentrations, exhibiting a strong “neutralizing” effect on diphtheria toxin. These findings suggest that this herb possesses significant detoxifying properties, addressing both chemical and microbial toxin-induced toxicity.

### 6.10. Others

The extract of *E. humifusa* has shown effectiveness in treating chronic urticaria, enhancing therapeutic outcomes, accelerating symptom relief, and demonstrating minimal adverse reactions, establishing it as a safe and reliable option. Additionally, the alcoholic extract significantly inhibits the contraction amplitude of rabbit small intestine smooth muscle, likely involving the activation of *α*-adrenergic receptors and inhibition of M cholinergic and H_1_ histamine receptors [45]. Research by Ma has indicated that EHH offers protective effects on ischemia-reperfusion in myocardial tissues [71]. Chang’s study explored the effect of *E. humifusa* polysaccharide on preventing ulcerative colitis through modulation of the intestinal microbiota [72].

Furthermore, *E. humifusa* polysaccharides are found to improve metabolic processes, enhance endurance, boost lactate dehydrogenase activity and glycogen levels, accelerate lactic acid clearance, reduce lactic acid buildup, inhibit excessive blood urea nitrogen levels post-exercise, delay fatigue, and enhance the body’s resilience to physical stress [73]. Analgesic properties of *E. humifusa* are also demonstrated in mice through a hotplate pain induction method [74]. Additionally, *E. humifusa* total polysaccharides have shown potential in preventing and treating animal diseases [75]. EHH is used as food in some countries, and toxicological studies have shown no significant toxicity from chronic use in rats, supporting its safety profile [5,76].

## 7. Summary and Perspectives

EHH has a long history of medicinal use and is showing encouraging results across diverse therapeutic areas, from treating diarrhea and dehydration to enhancing drug efficacy in combination therapies. Pharmacological research has uncovered its antidiabetic, antioxidant, antiviral, anti-rheumatoid arthritis, anticancer, and immunomodulatory properties, along with benefits for fatigue reduction, cardiovascular health, and animal disease prevention. Phytochemical analyses have identified numerous active compounds within EHH, including terpenoids, flavonoids, phenolic acids, alkaloids, steroids, and polysaccharides.

Before advancing its applications, comprehensive investigations into the chemical composition and pharmacological efficacy of EHH are essential. Although EHH has begun to be cultivated in certain areas, medicinal varieties in China are still largely sourced from the wild. The plant’s growth in diverse regions and climates results in variations in its quality and medicinal potency. To fully harness EHH’s pharmacological benefits, it is crucial to identify key bioactive markers and assess samples from multiple sources. Additionally, standardized cultivation bases should be established, with quality assessments conducted through advanced techniques such as NMR spectroscopy and HPLC-MS. By rigorously controlling market quality, TCM standards can be upheld, ensuring consistent therapeutic effects.

Moreover, 197 chemical constituents have been isolated from EHH, with terpenoids, flavonoids, and phenolic acids identified as particularly abundant. Future pharmacological research should continue focusing on these primary components while also employing more sophisticated methods and technologies to explore the effects of other components. Expanding this pharmacological understanding will provide a robust scientific foundation for clinical applications and broaden the therapeutic potential of EHH.

Historically, EHH has been valued for traditional medicinal uses, including its ability to clear heat and toxins, cool the blood and stop bleeding, eliminate dampness, and alleviate jaundice. In recent years, research and clinical applications have primarily focused on its effectiveness in treating diarrhea and dehydration. However, further exploration is required to understand other traditional effects, such as blood glucose reduction and anti-fatigue properties, by clarifying the underlying mechanisms and pharmacological actions to support broader clinical applications. This investigation will be crucial in validating EHH’s traditional uses.

As a plant with a rich history in traditional medicine, EHH has been widely employed for wellness and illness management. In this review, we examine the phytochemistry, pharmacological properties, and contemporary applications of EHH, aiming to deepen understanding of ongoing research and identify areas for further advancement. We also critically discuss potential future research directions for EHH, highlighting the need to investigate its traditional therapeutic effects and explore new therapeutic applications. These insights will provide strong scientific support for expanding EHH’s role in clinical interventions, enabling its use in a wider range of pharmacological and therapeutic contexts.

To deepen our understanding of EHH and advance its research, several key questions remain to be addressed. Firstly, although the value of EHH has gained increasing recognition, the quantity and quality of wild-harvested resources remain inconsistent. Thus, the development of artificial cultivation methods, along with the selection of disease-resistant varieties, is essential to improve both yield and quality. Secondly, while numerous studies have examined the phytochemistry of EHH, more focused attention is needed to lay the groundwork for future therapeutic applications.

Furthermore, given EHH’s extensive use in traditional medicine, systematic pharmacological studies, including an investigation into its pharmacodynamics and molecular mechanisms, are crucial to support its clinical application as both a TCM herb and a folk remedy in China. EHH represents a valuable resource. Future research should prioritize understanding its active compounds, mechanisms of action and the principles of its use in classical formulations, providing a solid theoretical foundation for broader applications and potential developments across various medical fields.

## Figures and Tables

**Table 1:** Triterpenoids in EHH.

NO.	Name	Molecular Formula	Molecular Weight	Source	References
**1**	(3*S*,4*S*,7*S*,9*R*)-4-methyl-3,7-dihydroxy-7(8→9) *abeo*-lanost-24(28)-en-8-one	C_30_H_50_O_3_	458.73	*E. maculata*	[12]
**2**	24-hydroperoxylanost-7,25-dien-3*β*-ol	C_30_H_50_O_3_	458.73	*E. maculata*	[12]
**3**	cycloart-23*Z*-en-3*β*, 25-diol	C_30_H_50_O_2_	442.73	*E. maculata*	[12]
**4**	3*β*-hydroxy-26-nor-9,19-cyclolanost-23-en-25-one	C_29_H_46_O_2_	426.69	*E. maculata*	[12]
**5**	cycloart-23en-3*β*,25-diol	C_30_H_50_O_2_	442.73	*E. maculata*	[12]
**6**	cycloeucalenol	C_30_H_50_O	426.73	*E. maculata*	[12]
**7**	(23*E*)-3*β*,25-dihydroxytirucalla-7,23-diene	C_30_H_50_O_2_	442.73	*E. maculata*	[12]
**8**	(23*Z*)-3*β*, 25-dihydroxy-tirucalla-7,23-diene	C_30_H_50_O_2_	442.73	*E. maculata*	[12]
**9**	obtusifoliol	C_30_H_50_O	426.73	*E. maculata*	[12]
**10**	4*α*, l4*α*-dimethyl-5*α*-ergosta-7,9(11), 24(28)-trien-3β-ol	C_30_H_48_O	424.71	*E. maculata*	[12]
**11**	gramisterol	C_30_H_50_O	426.73	*E. maculata*	[12]
**12**	urs-12-ene-3*β*, 11*α*-diol	C_30_H_50_O_2_	442.73	*E. maculata*	[12]
**13**	neoilexonol	C_30_H_48_O_2_	440.71	*E. maculata*	[12]
**14**	12-oleanene-3*β*,11*β*-diol	C_30_H_50_O_2_	442.73	*E. maculata*	[12]
**15**	(3*β*,15*α*, 16*α*)-15,16-epoxy, olean-12-en-3-ol	C_30_H_48_O_2_	440.71	*E. maculata*	[12]
**16**	multiflorenol	C_30_H_50_O	426.73	*E. maculata*	[12]
**17**	lupeol	C_30_H_50_O	426.73	*E. maculata*	[12]
**18**	3,4-seco-lupa-4(23),20(29)-dien-24-hydroxy-3-oic acid	C_30_H_48_O_3_	456.71	* E. humifusa *	[13]
**19**	lup-20(29)-ene-3,30-diol	C_30_H_50_O_2_	442.73	* E. humifusa *	[13]
**20**	24(*R*)-3,4-secocycloart-4(29),25-dien-24-hydroxy-3-oic acid	C_30_H_48_O_3_	456.71	* E. humifusa *	[13]
**21**	24(*S*)-3,4-seco-cycloart-4(29),25-dien-24-hydroxy-3-oic acid	C_30_H_48_O_3_	456.71	* E. humifusa *	[13]
**22**	23(*Z*)-cycloart-23-en-3,25-diol	C_30_H_50_O_2_	442.73	* E. humifusa *	[13]
**23**	24(*S*)-cycloart-25-en-3,24-diol	C_30_H_50_O_2_	442.73	* E. humifusa *	[13]
**24**	23(*E*)-cycloart-23-en-25-ethoxy-3-ol	C_32_H_54_O_2_	470.78	* E. humifusa *	[13]
**25**	3-hydroxy-4,14-dimethyl-5-ergosta-8,24(28)-dien-7-one	C_30_H_48_O_2_	440.71	* E. humifusa *	[13]
**26**	3-hydroxy-4,14-dimethyl-5-ergosta-8,24(28)-dien-7,11-dione	C_30_H_46_O_3_	454.70	* E. humifusa *	[13]
**27**	3-hydroxy-4,14-dimethyl-5-ergosta-7,9(11),24(28)-trien	C_30_H_48_O	424.71	* E. humifusa *	[13]
**28**	(3*S*,5*R*,7*R*,8*S*,10*S*,13*S*,14*S*,17*R*,18*R*,21*R*)−7*α*,8*α*-epoxyfern-9(11)-en-3*β*-ol	C_30_H_48_O_2_	440.71	* E. humifusa *	[14]
**29**	(3*R*,7*R*,8*S*,9*S*,13*S*,14*S*,17*R*,18*R*,21*R*)−7*α*,8*α*-epoxyadian-5(10)-en-3*α*-ol	C_30_H_48_O_2_	440.71	* E. humifusa *	[14]
**30**	fern-8(9)-en-3*β*-ol	C_30_H_50_O	426.73	* E. humifusa *	[14]
**31**	17*β*,21*β*-epoxyhopan-3*β*-ol	C_30_H_50_O_2_	442.73	* E. humifusa *	[14]
**32**	spiromaculatol A	C_31_H_52_O_3_	472.75	*E. maculate*	[15]
**33**	spiromaculatol B	C_31_H_52_O_3_	472.75	*E. maculate*	[15]
**34**	spiromaculatol C	C_30_H_50_O_3_	458.73	*E. maculate*	[15]
**35**	spiropedroxodiol	C_30_H_50_O_3_	458.73	*E. maculate*	[15]
**36**	spiroinonotsuoxodiol	C_30_H_50_O_3_	458.73	*E. maculate*	[15]
**37**	euphomaculatoid A	C_30_H_44_O_4_	468.68	*E. maculate*	[15]
**38**	euphomaculatoid B	C_30_H_46_O_4_	470.69	*E. maculate*	[15]
**39**	euphomaculatoid C	C_30_H_46_O_4_	470.69	*E. maculate*	[15]
**40**	euphomaculatoid D	C_30_H_46_O_3_	454.70	*E. maculate*	[15]
**41**	euphomaculatoid E	C_30_H_46_O_3_	454.70	*E. maculate*	[15]
**42**	3*β*, 7*α*-dihydroxy-4*α*,14*α*-dimethyl-5α-ergosta-8,24 (28)-dien-11-one	C_30_H_48_O_3_	456.71	*E. maculate*	[15]
**43**	3*β*-hydroxy-4*α*,14*α*-dimethyl-5*α*-ergosta-8,24 (28)-diene-7,11-dione	C_30_H_46_O_3_	454.70	*E. maculate*	[15]
**44**	euphomaculatoid F	C_29_H_44_O_4_	456.67	*E. maculate*	[15]
**45**	euphomaculatoid G	C_29_H_42_O_4_	454.65	*E. maculate*	[15]
**46**	euphomaculatoid H	C_30_H_48_O_3_	456.71	*E. maculate*	[15]
**47**	3*β*,11*β*-3,11-dihydroxylanosta-8,24-dien-7-one	C_30_H_48_O_3_	456.71	*E. maculate*	[15]

**Table 2:** Flavonoids in EHH.

NO.	Name	Molecular Formula	Molecular Weight	Source	References
**48**	luteolin	C_15_H_10_O_6_	286.24	*E. humifusa*	[16,25]
**49**	apigenin	C_15_H_10_O_5_	270.24	*E. humifusa*	[16]
**50**	luteolin-7-*O*-(6″-*O*-trans-feruloyl)-*β*-*D*-glucopyranoside	C_31_H_28_O_14_	624.55	*E. humifusa*	[16]
**51**	luteolin-7-*O*-(6″-*O*-coumaroyl)-*β*-*D*-glucopyranoside	C_25_H_26_O_14_	550.47	*E. humifusa*	[16]
**52**	apigenin-7-*O*-*β*-*D*-lutinoside	C_27_H_30_O_14_	578.52	*E. humifusa*	[16]
**53**	apigenin-7-*O*-*β*-*D*-apiofuranosyl(1→2)-*β*-*D*-glucopyranoside	C_30_H_26_O_13_	594.14	*E. humifusa*	[16]
**54**	6,8-di-*C*-*β*-*D*-glucopyranosyl apigenin	C_27_H_30_O_15_	594.52	*E. humifusa*	[16]
**55**	apigenin-7-*O*-(6″-*O*-galloyl)-*β*-*D*-glucopyranoside	C_28_H_24_O_14_	584.49	*E. humifusa*	[16]
**56**	quercetin-3-*O*-*β*-*D*-galactoside	C_21_H_20_O_12_	464.38	*E. humifusa*	[16]
**57**	quercetin-3-*O*-*β*-*D*-glucopyranoside	C_21_H_20_O_12_	464.38	*E. humifusa*	[16]
**58**	hesperidin	C_29_H_36_O_14_	608.59	*E. humifusa*	[16]
**59**	quercetin-7-*O*-*β*-*D*-glucopyranoside	C_21_H_20_O_12_	464.38	*E. humifusa*	[20]
**60**	quercetin-3-*O*-*α*-*L*-rhamnosyl(1→6)-*β*-*D*-galactoside	C_27_H_30_O_16_	610.52	*E. humifusa*	[20]
**61**	kaempferol-3-*O*-*β*-*D*-glucopyranoside	C_21_H_20_O_11_	448.38	*E. humifusa*	[20]
**62**	quercetin	C_15_H_10_O_7_	302.24	*E. humifusa*	[20]
**63**	kaempferol	C_15_H_10_O_6_	286.24	*E. humifusa*	[21]
**64**	isorhoifolin	C_27_H_30_O_14_	578.52	*E. humifusa*	[22]
**65**	kaempferol-3-*O*-*α*-*L*-arabinoforanoside	C_20_H_18_O_10_	418.35	*E. humifusa*	[22]
**66**	apigenin-7-*O*-glucoside	C_21_H_20_O_10_	432.38	*E. humifusa*	[23]
**67**	luteolin-7-*O*-glucoside	C_21_H_20_O_11_	448.38	*E. humifusa*	[23]
**68**	quercetin-3-*O*-arabinoside	C_20_H_18_O_11_	434.35	*E. humifusa*	[22]
**69**	luteolin-7-*O*-*β*-*D*-glucopyranoside	C_21_H_20_O_11_	448.38	*E. humifusa*	[24]
**70**	apigenin-7-*O*-*β*-*D*-glucopyranoside	C_21_H_20_O_10_	432.38	*E. humifusa*	[24]
**71**	quercetin-3-*O*-(2″,3″-di-*O*-galloyl)-*β*-*D*-glucopyranoside	C_28_H_24_O_16_	616.48	*E. humifusa*	[24]
**72**	quercetin-3-*O*-(6″-*O*-galloyl)-glucopyranoside	C_28_H_24_O_16_	616.48	*E. humifusa*	[24]
**73**	quercetin-3-*O*-{rhamnosyl-(1→6)-[xylosyl-(1→2)]-galactoside}	C_32_H_38_O_20_	742.64	*E. humifusa*	[24]
**74**	quercetin-3-*O*-*β*-*D*-xylosyl-(1→2)-*β*-*D*-glucopyranoside	C_26_H_28_O_16_	596.49	*E. humifusa*	[24]
**75**	nicotiflorin	C_27_H_30_O_15_	594.52	*E. humifusa*	[24]
**76**	kaempferol-3-*O*-(6″-*O*-galloyl)-*β*-*D*-galactoside	C_28_H_24_O_15_	600.49	*E. humifusa*	[24]
**77**	isomyricitrin	C_21_H_20_O_13_	480.38	*E. humifusa*	[24]
**78**	phlorizin	C_21_H_24_O_10_	436.41	*E. humifusa*	[24]
**79**	quercetin-3-*O*-*α*-*L*-arabinofuranoside	C_20_H_18_O_11_	434.35	*E. humifusa*	[3]
**80**	8-hydroxyluteolin	C_15_H_10_O_7_	302.24	*E. humifusa*	[25]
**81**	paratocarpin E	C_25_H_28_O_4_	392.50	*E. humifusa*	[25]
**82**	quercetin-3-*O*-apiosyl (1–2) galactoside	C_26_H_28_O_16_	596.49	*E. humifusa*	[19]
**83**	quercetin-3-*O*-glucoside	C_21_H_20_O_11_	448.38	*E. humifusa*	[19]
**84**	astragalin	C_21_H_20_O_12_	464.38	*E. humifusa*	[3,19]
**85**	vitexin	C_21_H_20_O_10_	432.38	*E. humifusa*	[19]
**86**	isoquercitrin 6″-*O*-gallate	C_28_H_24_O_16_	616.48	*E. maculata*	[18]
**87**	astragalin 6″-*O*-gallate	C_28_H_24_O_15_	600.48	*E. maculata*	[18]
**88**	avicularin	C_20_H_18_O_11_	434.35	*E. maculata*	[18]
**89**	juglanin	C_20_H_18_O_10_	418.35	*E. maculata*	[18]
**90**	isoquercitrin	C_21_H_20_O_12_	464.38	*E. maculata*	[18]
**91**	hyperoside	C_21_H_20_O_12_	464.38	*E. maculata*	[18]
**92**	astragalin 2″,3″-*O*-digallate	C_36_H_30_O_19_	766.62	*E. humifusa*	[4]
**93**	astragalin 2″-*O*-gallate	C_29_H_26_O_15_	614.51	*E. humifusa*	[4]
**94**	isoquercitin 2″-*O*-gallate	C_29_H_26_O_16_	630.51	*E. humifusa*	[4]
**95**	quercitrin 2″-*O*-gallate	C_29_H_26_O_15_	614.51	*E. humifusa*	[4]
**96**	hyperin	C_21_H_20_O_12_	464.38	*E. humifusa*	[4]
**97**	rutin	C_27_H_30_O_16_	616.49	*E. humifusa*	[4]

**Table 3:** Phenolic acids and tannins in EHH.

NO.	Name	Molecular Formula	Molecular Weight	Source	References
**98**	gallic acid	C_7_H_6_O_5_	170.12	*E. humifusa*	[18]
**99**	methyl gallate	C_8_H_8_O_5_	184.15	*E. humifusa*	[18]
**100**	1,3,6-tri-*O*-galloyl-2-*O*-brevifolincarboxyl-*β*-*D*-glucose	C_40_H_30_O_25_	910.66	*E. humifusa*	[26]
**101**	tellimagrandin I	C_35_H_28_O_21_	784.11	*E. humifusa*	[26]
**102**	excoecarianin	C_82_H_56_O_53_	1888.17	*E. humifusa*	[26]
**103**	ethyl gallate	C_9_H_10_O_5_	198.17	*E. humifusa*	[16]
**104**	phyllanthussin E methyl ester	C_14_H_10_O_8_	306.4	*E. humifusa*	[16]
**105**	humifusaone	C_12_H_10_O_7_	266.21	*E. humifusa*	[16]
**106**	valoneaic aciddilactone	C_21_H_10_O_13_	470.30	*E. humifusa*	[27]
**107**	eumaculin E	C_41_H_32_O_28_	972.68	*E. maculata*	[16]
**108**	dehydropicrorhiza acid methyl diester	C_16_H_12_O_10_	364.26	*E. humifusa*	[4]
**109**	methylsyringin	C_18_H_26_O_9_	386.16	*E. humifusa*	[4]
**110**	syringin	C_17_H_24_O_8_	356.15	*E. humifusa*	[4]
**111**	methylconiferin	C_17_H_24_O_9_	372.14	*E. humifusa*	[4]
**112**	sphaerophyside SC	C_16_H_22_O_7_	326.35	*E. humifusa*	[4]
**113**	1,2,3-tri-*O*-galloyl-*β*-*D*-glucose	C_27_H_24_O_18_	636.47	*E. maculata*	[28]
**114**	granatin B	C_42_H_30_O_26_	950.68	*E. maculata*	[27]
**115**	ellagic acid-4-*O*-*β*-*D*-glucopyranoside	C_20_H_16_O_13_	464.34	*E. humifusa*	[26]
**116**	3,3′-di-*O*-methyl ellagic acid-4-*O*-*β*-*D*-glucopyranoside	C_22_H_20_O_13_	492.39	*E. humifusa*	[22]
**117**	brevifolin carboxylic acid	C_12_H_8_O_6_	248.19	*E. humifusa*	[4]
**118**	methyl brevifolin carboxylate	C_14_H_10_O_8_	306.23	*E. humifusa*	[16]
**119**	ethyl brevifolin carboxylate	C_15_H_12_O_8_	320.25	*E. humifusa*	[16]
**120**	sanguisorbicacid dilactone	C_21_H_10_O_13_	470.30	*E. humifusa*	[16]
**121**	7”-ethyl-sanguisorbic acid dilactone	C_23_H_14_O_13_	498.35	*E. humifusa*	[16]
**122**	1-*O*-methyl-6-*O*-p-digalloyl-*α*-*D*-glucopyranoside	C_21_H_22_O_12_	466.40	*E. humifusa*	[12]
**123**	1-*O*-ethyl-6-*O*-p-digalloyl-*α-D*-glucopyranoside	C_22_H_24_O_12_	480.42	*E. humifusa*	[16]
**124**	chebulanin	C_27_H_24_O_19_	652.47	*E. humifusa*	[16]
**125**	furosin	C_27_H_22_O_19_	650.45	*. humifusa*	[16]
**126**	1,3,4,6-tetra-*O*-galloyl-*β*-*D*-glucose	C_27_H_24_O_18_	636.47	*E. humifusa*	[26]
**127**	Euphormisin M3	C_41_H_32_O_26_	940.68	*E. humifusa*	[26]
**128**	1,2,4,6-tetra-*O*-galloyl-*β*-*D*-glucose	C_34_H_28_O_22_	788.58	*E. humifusa*	[26]
**129**	1,2,6-tri-*O*-galloyl-*β*-*D*-glucose	C_27_H_24_O_18_	636.47	*E. humifusa*	[26]
**130**	1,3,4,6-tetra-*O*-galloyl-*β*-*D*-glucose	C_34_H_28_O_22_	788.58	*E. humifusa*	[26]
**131**	2,4,6-tri-*O*-galloyl-*D*-glucose	C_27_H_24_O_18_	636.47	*E. humifusa*	[26]
**132**	3,4,6-tri-*O*-galloyl-*D*-glucose	C_27_H_24_O_18_	636.47	*E. humifusa*	[26]
**133**	1,3,6-tri-*O*-galloyl-*D*-glucose	C_27_H_24_O_18_	636.47	*E. humifusa*	[26]
**134**	euphormisin M2	C_41_H_30_O_25_	922.67	*E. maculata*	[28]
**135**	eumaculinA	C_69_H_54_O_43_	1571.15	*E. maculata*	[28]
**136**	geraniin	C_41_H_28_O_27_	952.65	*E. maculata*	[28]
**137**	euphorbin B	C_83_H_60_O_52_	1889.34	*E. maculata*	[28]
**138**	euphorbin A	C_125_H_92_O_77_	2826.03	*E. maculata*	[28]
**139**	chebulagic acid	C_41_H_30_O_27_	954.66	*E. maculata*	[28]
**140**	mallotusinin	C_41_H_26_O_25_	918.63	*E. maculata*	[27]
**141**	corilagin	C_27_H_22_O_18_	634.46	*E. maculata*	[27]
**142**	1-*O*-ethyl-3,6-*O*-(*R*)-hexahydroxydiphenoyl-(1C4)-*β*-*D*-glucose	C_22_H_22_O_14_	510.40	*E. maculata*	[27]
**143**	1,3,4,6-tetra-*O*-galloyl-*β*-*D*-glucose	C_41_H_28_O_27_	952.65	*E. maculata*	[27]
**144**	tercatain	C_34_H_26_O_22_	786.56	*E. maculata*	[27]
**145**	eumaculin B	C_69_H_54_O_43_	1571.15	*E. maculata*	[27]
**146**	eumaculin D	C_69_H_54_O_43_	1571.15	*E. maculata*	[27]
**147**	protocatechuic acid	C_7_H_6_O_4_	154.12	*E. humifusa*	[29]
**148**	*cis*-caffeic acid	C_9_H_8_O_4_	180.16	*E. humifusa*	[29]
**149**	*trans*-caffeic acid	C_9_H_8_O_4_	180.16	*E. humifusa*	[29]
**150**	rosmarinic	C_18_H_16_O_8_	360.32	*E. humifusa*	[30]
**151**	euphorbinoside	C_27_H_32_O_14_	580.54	*E. humifusa*	[4]
**152**	benzyl *β*-D-ribofuranoside	C_12_H_16_O_5_	240.26	*E. humifusa*	[4]

**Table 4:** The alkaloids in EHH.

NO.	Name	Molecular Formula	Molecular Weight	Source	References
**153**	5-*β*-methoxy-4*β*-hydroxy-3-methylene-*α*-pyrrolidinone	C_6_H_9_NO_3_	143.14	*E. humifusa*	[22]
**154**	5-*β*-methoxy-4*α*-hydroxy-3-methylene-*α*-pyrrolidinone	C_6_H_9_NO_3_	143.14	*E. humifusa*	[22]
**155**	5*β*-butoxy-4*α*-hydroxy-3-methylene-*α*-pyrrolidinone	C_9_H_15_NO_3_	185.22	*E. humifusa*	[22]
**156**	3-(2-hydroxyethyl)-5-(1-*O*-glucopyranosyloxy)-indole	C_16_H_21_NO_7_	339.34	*E. humifusa*	[22]
**157**	1-(2′,3′,4′,5′-tetrahydroxypentyl)-6,7-dimethyl-guinoxaline-2,3-(1H,4H)-dione	C_15_H_20_N_2_O_6_	324.33	*E. humifusa*	[16]
**158**	uinoxadione	C_8_H_6_N_2_O	146.15	*E. humifusa*	[26]
**159**	(−)-neoechinulin A	C_19_H_21_N_3_O_2_	323.40	*E. humifusa*	[29]

**Table 5:** The sterols in EHH.

NO.	Name	Molecular Formula	Molecular Weight	Source	References
**160**	*β*-sitosterol	C_29_H_50_O	414.72	*E. humifusa*	[24]
**161**	*β*-daucosterol	C_35_H_60_O_6_	576.85	*E. humifusa*	[24]
**162**	stigmaster-5-ene-3-*O*-(6-linoyl-114yl)-*β*-*D*-glucopyranoside	C_35_H_60_O_7_	592.85	*E. humifusa*	[24]
**163**	7*β*-hydroxy-sitosterol	C_29_H_50_O_2_	430.72	*E. humifusa*	[24]

**Table 6:** The lactones and coumarins in EHH.

NO.	Name	Molecular Formula	Molecular Weight	Source	References
**164**	scopoletin	C_10_H_8_O_4_	192.17	*E. humifusa*	[24]
**165**	umbelliferone	C_9_H_6_O_3_	162.14	*E. humifusa*	[23]
**166**	7-methoxy-6-hydroxyl-coumarin	C_10_H_8_O_4_	192.17	*E. humifusa*	[13]
**167**	esculetin	C_9_H_6_O_4_	178.14	*E. humifusa*	[29]
**168**	5-methoxyscopoletin	C_11_H_10_O_5_	222.20	*E. humifusa*	[29]
**169**	isofraxidin	C_11_H_10_O_5_	222.20	*E. humifusa*	[29]
**170**	ethyl brevifolincarboxylate	C_15_H_12_O_8_	320.25	*E. humifusa*	[13]

**Table 7:** Other compounds in EHH.

NO.	Name	Molecular Formula	Molecular Weight	Source	References
**171**	*α*-pyrone	C_6_H_6_O_2_	110.04	*E. humifusa*	[31]
**172**	*γ*-pyrone	C_5_H_4_O_2_	96.09	*E. humifusa*	[31]
**173**	2-methoxy-4-vinylphenol	C_9_H_10_O_2_	150.18	*E. humifusa*	[31]
**174**	desogestrel	C_15_H_24_	204.36	*E. humifusa*	[31]
**175**	*α*-ionone	C_13_H_20_O	192.30	*E. humifusa*	[31]
**176**	dihydroactinidiolide	C_11_H_16_O_2_	180.25	*E. humifusa*	[31]
**177**	lauric acid	C_12_H_24_O_2_	200.32	*E. humifusa*	[31]
**178**	isononyl phthalate	C_26_H_48_O_4_	424.67	*E. humifusa*	[31]
**179**	methyl hexadecanoate	C_18_H_36_O	268.49	*E. humifusa*	[31]
**]180**	dibutyl phthalate	C_16_H_28_O_4_	284.40	*E. humifusa*	[31]
**181**	palmitic acid	C_16_H_32_O_2_	256.43	*E. humifusa*	[31]
**182**	methyl linoleate	C_19_H_34_O_2_	294.48	*E. humifusa*	[31]
**183**	9,12,15-octadecatrienoic acid, methyl ester	C_21_H_36_O_2_	320.52	*E. humifusa*	[31]
**184**	phytol	C_20_H_40_O	296.54	*E. humifusa*	[31]
**185**	linoleic acid	C_18_H_30_O_2_	278.44	*E. humifusa*	[31]
**186**	*α*-linolenic acid	C_24_H_42_O_2_	362.60	*E. humifusa*	[31]
**187**	humionoactoside A	C_38_H_60_O_9_	660.89	*E. humifusa*	[32]
**188**	(2*S*)-3-*O*-octadeca-9*Z*,12*Z*,15*Z*-trienoylglyceryl-*O*-*β*-D-galactopyranoside	C_28_H_48_O_9_	528.68	*E. humifusa*	[32]
**189**	gingerglycolipid A	C_34_H_58_O_14_	690.82	*E. humifusa*	[32]
**190**	6′-*O*-linolenoylsucrose	C_31_H_52_O_12_	616.75	*E. humifusa*	[32]
**191**	(5*Z*)-nonenoic acid	C_9_H_16_O_2_	156.23	*E. humifusa*	[29]
**192**	(5*Z*)-undecenoic acid	C_10_H_18_O_2_	170.25	*E. humifusa*	[29]
**193**	corchoionol C	C_13_H_20_O_3_	224.30	*E. humifusa*	[29]
**194**	vomifoliol	C_13_H_20_O_3_	224.30	*E. humifusa*	[29]
**195**	(−)-phaseic acid	C_15_H_20_O_5_	280.32	*E. humifusa*	[29]
**196**	isololiiolide	C_11_H_16_O_3_	196.25	*E. humifusa*	[29]
**197**	(4*S*)-*α*-terpineol 8-*O*-[*α*-L-arabinopyranosyl-(1→6)-*β*-D-glucopyranoside]	C_21_H_36_O_10_	448.51	*E. humifusa*	[4]

## Data Availability

No new data were created or analyzed in this study.

## Abbreviations

AP-1	Activator protein 1
CHB	Chronic hepatitis B
CNKI	China National Knowledge Infrastructure
CXCL1	(C-X-C motif) ligand 1 protein
CXCL5	(C-X-C motif) ligand 5 protein
DGGlc	2,3-Digalloyglucosyl
DJC	Dijincao
DKD	Diabetic kidney disease
DM	Diabetes mellitus
EHH	Euphorbiae Humifusae Herba
ELISA	Enzyme-linked immunosorbent assay
FvGFR3	Fibroblast growth factor receptors
Gal	Galactosyl
GGlc	2-Galloyglucosyl
GRham	2-Galloylrhamosyl
HBV	Hepatitis B virus
IL-1	Interleukin-1
IL-10	Interleukin-10
IL-17	Interleukin-17
IL-1*β*	Interleukin-1*β*
IL-2	Interleukin-2
IL-6	Interleukin-6
IL-8	Interleukin-8
IP-10	Inducible Protein 10
LTB4	Leukotriene B4
MAPK	Mitogen-activated protein kinase
MCP-1	Monocyte chemoattractant protein-1
MDA	Malondialdehyde
MMP9	Matrix metalloproteinase 9
NF-*κ*B	Nuclear factor-*κ*B
PGE2	Prostaglandin E2
PI3K	Phosphatidylinositol 3-kinase
PKC	Protein kinase C
RA	Rheumatoid arthritis
Rut	Rutinosyl
SOD	Superoxide dismutase
SPF	Specific pathogen-free
TCM	Traditional Chinese medicine
TGF-*β*	Transforming growth factor-*β*
Th17	T helper cell 17
TNF-α	Tumor necrosis factor-*α*
VZV	Varicella-zoster virus
WHO	World Health Organization
